# Targeting Ion Channels and Purkinje Neuron Intrinsic Membrane Excitability as a Therapeutic Strategy for Cerebellar Ataxia

**DOI:** 10.3390/life13061350

**Published:** 2023-06-08

**Authors:** Haoran Huang, Vikram G. Shakkottai

**Affiliations:** 1 Medical Scientist Training Program, The Ohio State University College of Medicine, Columbus, OH 43210, USA; haoran.huang@osumc.edu; 2 Department of Neurology, The University of Texas Southwestern Medical Center, Dallas, TX 75390, USA

**Keywords:** cerebellar ataxia, Purkinje neuron, intrinsic membrane excitability, ion channels, therapy

## Abstract

In degenerative neurological disorders such as Parkinson’s disease, a convergence of widely varying insults results in a loss of dopaminergic neurons and, thus, the motor symptoms of the disease. Dopamine replacement therapy with agents such as levodopa is a mainstay of therapy. Cerebellar ataxias, a heterogeneous group of currently untreatable conditions, have not been identified to have a shared physiology that is a target of therapy. In this review, we propose that perturbations in cerebellar Purkinje neuron intrinsic membrane excitability, a result of ion channel dysregulation, is a common pathophysiologic mechanism that drives motor impairment and vulnerability to degeneration in cerebellar ataxias of widely differing genetic etiologies. We further propose that treatments aimed at restoring Purkinje neuron intrinsic membrane excitability have the potential to be a shared therapy in cerebellar ataxia akin to levodopa for Parkinson’s disease.

## 1. Introduction

The cerebellar ataxias are a group of clinically heterogeneous movement disorders that primarily affect the cerebellum. The causes of cerebellar ataxia are diverse, but patients share similar clinical features, including abnormal gait, impaired balance, poor coordination of limbs, and speech impairment. Despite similar clinical manifestations, a shared treatment for cerebellar ataxia akin to levodopa for Parkinson’s disease has not been identified.

Among all types of cerebellar ataxias, spinocerebellar ataxias (SCAs) are a major subgroup of dominantly inherited neurological disorders causing cerebellar impairment. Currently, more than 40 genetic mutations leading to SCAs have been identified [1]. Interestingly, though the disease-causing genes vary, Purkinje neuron pathophysiology is a shared feature of the disease.

Purkinje neurons are located in the cerebellar cortex and integrate all the input into the cerebellum [2]. Cerebellar atrophy and Purkinje neuron degeneration are shared features of cerebellar ataxia [3,4]. One unique feature of Purkinje neurons is that they exhibit autonomous spiking independent of synaptic stimulation. This pacemaking ability of Purkinje neurons is thought to play a vital role in motor coordination as those disturbances in Purkinje neuron firing significantly impair motor function in mouse models of SCA [5,6,7,8,9,10,11,12]. More importantly, in these mouse models of ataxia, rescuing Purkinje neuron firing abnormalities improves motor performance (additional models reviewed in [13]).

The average firing frequency of Purkinje neurons is around 40 Hz, with an unvarying inter-spike interval duration under resting conditions. This regularity is primarily maintained with precisely controlled activities of ion channels [14]. In brief, activation of sodium channels, primarily Nav1.6 but also Nav1.1, depolarizes the Purkinje neuron plasma membrane. Activation of voltage-gated sodium channels depolarizes the membrane to a threshold to initiate the action potential. Voltage-gated potassium channels are then activated, mediating the repolarization phase of the action potential. The depolarization also activates voltage-gated calcium channels, primarily Cav2.1 and Cav3.1, allowing calcium into Purkinje neurons. These calcium channels are coupled to calcium-activated potassium channels, which generate an outward potassium current upon calcium entry and hyperpolarize the Purkinje neuron membrane to generate the after-hyperpolarization or AHP. The major calcium-activated potassium channels that are responsible for generating the AHP in Purkinje neurons are large-conductance calcium-activated potassium (BK) channels and small-conductance calcium-activated potassium type 2 (SK2) channels. The AHP is essential for the initiation of the subsequent action potential by allowing sodium channels to recover from inactivation and preventing the cell from undergoing a depolarization block. Importantly, numerous studies have shown that perturbations of expression and function of these ion channels alter the intrinsic membrane excitability of Purkinje neurons, leading to abnormalities in Purkinje neuron firing (reviewed in [13,15]). In mouse models of the disease, the abnormalities in Purkinje neuron spiking underlie motor dysfunction, at least in the early stages of the disease. Given the important role of ion channels in regulating proper Purkinje neuron physiology, targeting ion channel function and thereby modulating Purkinje neuron intrinsic membrane excitability holds promise as a therapeutic strategy for cerebellar ataxia.

It is worth considering another degenerative disorder, namely Parkinson’s disease when evaluating whether a shared treatment strategy exists for cerebellar ataxia. Parkinson’s disease is a collection of inherited and sporadic disorders defined by motor impairments of rigidity, tremors, bradykinesia, and postural-gait changes. Most importantly, in spite of diverse potential molecular mechanisms for disease, motor impairment is sensitive to improvement with dopamine replacement therapy, with levodopa being a mainstay of therapy. The cerebellar ataxias, too, are clinically defined by the motor impairment that they produce. In this review, we summarize ion channel gene mutations that result in human cerebellar ataxia. We speculate that targeting ion channels pharmacologically to correct aberrant Purkinje neuron intrinsic membrane excitability holds promise in the treatment of cerebellar ataxia more widely.

## 2. Ion Channel Gene Mutations That Cause Ataxia

### 2.1. Voltage-Gated Sodium Channels

Sodium channels, Nav1.6 and Nav1.1, are important in generating the action potential upstroke in cerebellar Purkinje neurons [16,17]. Nav1.6 is encoded by the gene *SCN8A*, gain-of-function mutations of which are associated with epileptic encephalopathy with ataxia (Table 1) [18]. In mice, partial or complete loss-of-function mutations of *Scn8a* result in motor impairment, symptoms of which also include ataxia [19]. Cerebellar Purkinje neurons of *Scna8a* null mice display reduced repetitive firing [20]. Ataxia is, therefore, associated with *SCN8A* mutations in both human and mouse models.

Dravet syndrome is caused by mutations in *SCN1A*, the gene that encodes Nav1.1 (Table 1) [21]. The clinical manifestations of Dravet syndrome include seizures, cognitive impairment, motor deficits, and ataxia [22]. Knockout of Nav1.1 in mice also results in an ataxic phenotype [16]. Recently, it has been shown that either activating Nav1.1 or blocking Nav1.6 can reduce seizure occurrence in a zebrafish model of Dravet syndrome [23], demonstrating the therapeutic potential of targeting these sodium channels.

Defects in the sodium channel, Nav1.6, are also associated with SCA27 in humans. SCA27 results from mutations in the *FGF14* gene, which encodes fibroblast growth factor 14 (FGF14) [24]. *Ffg14* null mice exhibit an ataxic phenotype that resembles SCA27 patients [25]. Spontaneous Purkinje neuron firing in the *Fgf14* null mice is attenuated, accompanied by a negative shift of membrane potential [26]. It has been shown that spontaneous firing of Purkinje neurons requires intracellular FGF14 (iFGF14), and expressing iFGF14 specifically in Purkinje neurons of *Fgf14* null mice restores spontaneous firing and improves motor performance [27]. In addition, FGF14 interacts with Nav1.6 [28], and the level of Nav1.6 is reduced in Purkinje neurons of FGF14 null mice [26]. These data suggest that the interaction between FGF14 and Nav1.6 is crucial for Nav1.6 expression and function in the cerebellum [26].

Recently, a new genetic cause of late-onset ataxia has been uncovered. This new type of ataxia, termed late-onset SCA27B, is associated with an intronic GAA repeat expansion in *FGF14* [29]. Patients with SCA27B display both episodic and progressive ataxia, with an average age of onset of 55 years [29]. Examination of brain tissue from patients reveals cerebellar atrophy with significant depletion of Purkinje neurons and reduction in FGF14 expression (Table 1). Given the prior data in *Fgf14* knockout mice, it is likely that reduced FGF14 level induced reduction in Nav1.6 expression contributes to disease symptoms.

**Table 1:** Summary of ion channels and their associated disease and the cerebellar pathology that results.

Types	Ion Channel	Disease	Cerebellar Pathology
Voltage-gated sodium channels	Nav1.1	Dravet syndrome	Cerebellar atrophy on MRI; Cerebellar atrophy with loss of Purkinje neuron on post-mortem tissue [30].
	Nav1.6	Epileptic encephalopathy	Cerebellar atrophy on MRI in one patient [31].
		SCA27B	Severe cerebellar vermis atrophy on MRI and post-mortem tissue. Loss of Purkinje neurons on post-mortem tissue [29].
Voltage-gated potassium channels	Kv1.1	EA1	No cerebellar pathology was reported.
	Kv1.2	Ataxia associated with epileptic encephalopathy	Cerebellar atrophy on MRI in a subset of patients [32].
	Kv1.6	SCA3	See above.
	Kv3.3	SCA13	Cerebellar atrophy on MRI [33].
		SCA3	Mild cerebellar atrophy with enlarged 4th ventricle [4]; Degeneration of the cerebellar fastigial nucleus [3].
		SCA1,2	See above.
	Kv4.3	SCA19, 22	Severe Purkinje neuron degeneration in cerebellar autopsy. Mild cerebellar atrophy on MRI in some patients [34,35].
		SCA1	See above.
Calcium-activated potassium channels	BK	Liang-Wang syndrome	Cerebral atrophy involving the vermis and hemisphere on MRI [36,37].
		SCA1	Global cerebellar volume loss on MRI [4]. Cerebellar atrophy on biopsy and degeneration of cerebellar Purkinje neurons, and the cerebellar fastigial nucleus [3].
		SCA2	Cerebellar atrophy on MRI [38]; Global cerebellar volume loss involving both the vermis and cerebellar hemispheres on MRI [4]; Degeneration of cerebellar Purkinje neurons and the cerebellar fastigial nucleus [3].
		SCA7	Cerebellar atrophy mainly involves the superior part of the vermis on MRI [4]; Degeneration of cerebellar Purkinje neurons and the cerebellar fastigial nucleus [3].
	SK2	Dominant neurodevelopmental movement disorders; autosomal-dominant tremulous myoclonus-dystonia	Cerebellar atrophy on MRI in one case [39] (Mochel, personal communication)
Voltage-gated calcium channels	Cav2.1	SCA6	Cerebellar atrophy involving the vermis and the hemisphere [4]; Degeneration of cerebellar Purkinje neurons.
		EA2	Cerebellar vermis atrophy on MRI [40].
	Cav3.1	SCA42	Cerebellar atrophy on MRI [41,42].
		SCA1,2,7	See above.
Other calcium channels and calcium pumps	TRPC3	SCA41	Mild cerebellar vermis atrophy on MRI [43].
	IP_3_R1	SCA15	Cerebellar atrophy on MRI and CT [44].
		SCA29	Cerebellar atrophy on MRI [45].
		SCA2, 3	See above.
	PMCA2	Congenital cerebellar ataxia	Cerebellar atrophy on MRI [46].
	PMCA3	X-linked congenital cerebellar ataxia	Volume loss of cerebellar hemisphere and vermis on MRI [47].

### 2.2. Voltage-Gated Potassium Channels

#### 2.2.1. Kv1 Channel Family

Kv1 channels are a group of voltage-gated potassium channels that play an essential role in regulating membrane excitability in neurons. It has been shown that Kv1.1, Kv1.2, Kv1.3, Kv1.5, and Kv1.6 are highly enriched in the rat cerebellum, with higher densities of Kv1.1 and Kv1.2 in the terminal region of Purkinje neurons and higher densities of Kv1.5 and Kv1.6 in the soma [48]. Among them, Kv1.1, Kv1.2, and Kv1.6 expression changes are reported to be associated with ataxia [49].

##### Kv1.1

Mutations in Kv1.1 channels result in Episodic ataxia type 1 (EA1), which is characterized by episodes of loss of motor coordination and balance in addition to prominent muscle spasms involving the head and extremities (Table 1) [50]. To date, a number of gene mutations in *KCNA1*, the gene encoding Kv1.1 channels, have been identified in patients with EA1. Functional studies of mutated Kv1.1 reveal loss-of-function effects, including a reduction in current, abnormal gating properties, and/or a positive shift of voltage dependence [51].

Gain-of-function mutations in *KCNA1* have also been reported. A patient with a p. A261T Kv1.1 variant presented with mild, childhood-onset focal epilepsy without ataxia [52]. Another patient with a p.L296F mutation was described to display recurrent onset of seizures [53]. Both mutations cause an alteration in the voltage dependence of mutant Kv1.1 channel activation, resulting in the channel opening at a more hyperpolarized state compared to wild-type channels. In addition, treating the patient with the p.L296F variant with 4-aminopyridine, a non-specific potassium channel blocker, improved motor impairment.

##### Kv1.2

The Kv1.2 channel is encoded by the gene *KCNA2*. De novo mutations in *KCNA2* are associated with epileptic encephalopathy, with about half of the patients with *KCNA2* mutations showing symptoms of ataxia and atrophy of the cerebellum (Table 1) [32]. Interestingly, both gain- and loss-of-function mutations of *KCNA2* are associated with encephalopathy. Specifically, p.R297Q, p.L298F, and p.E157K variants cause an increase in current amplitude and a more hyperpolarized voltage dependence of activation [32]. In contrast, p.I263T and p.P405L variants result in a loss-of-function, characterized by a significant reduction in current amplitude [54]. Furthermore, p.L290R, p.L293H, p.L328V, and T374A variants demonstrate both gain- and loss-of-function effects, resulting in the permanent opening of the channel with a negative voltage shift of inactivation. Patients harboring either a gain-of-function mutation or a combination of gain- and loss-of-function mutations in *KCNA2* are more likely to develop ataxia and cerebellar atrophy [32,54]. 4-aminopridine has been shown to improve symptoms in patients with gain-of-function *KCNA2* encephalopathy [55].

In mice, a p.I402T mutation in *Kcna2* results in chronic motor incoordination. Patch clamp recordings from cerebellar slices of these mice reveal reduced Purkinje neuron firing frequency. In addition, the protein levels of both Kv1.2 and Kv1.1 channels are reduced. Increasing the expression of the Kv1.2 channel partially rescues impaired motor coordination [56].

##### Kv1.6

The Kv1.6 channel is encoded by the gene *KCNA6*. A recent study identified de novo gain-of-function mutations in *KCNA6* in patients with epilepsy [57]. Mutant Kv1.6 channels display slowed channel closing time and a negative shift in deactivation voltage. Reduced transcript levels of *Kcna6* are associated with increased Purkinje neuron intrinsic membrane excitability in SCA3 mice (Table 1). Treatment with antisense oligonucleotides targeting mutant *Atxn3* rescues the reduction in *Kcna6* transcript levels in SCA3 mice [49].

#### 2.2.2. Kv3.3

Kv3.3 is a voltage-dependent potassium channel encoded by the gene *KCNC3*. Mutations in *KCNC3* cause SCA13 in humans, which is characterized by cerebellar degeneration, impaired motor function, and abnormal auditory processing (Table 1) [58]. The age of disease onset in SCA13 patients ranges from early after birth to middle age. *Kcnc3* knockout mice exhibit a reduction in Purkinje neuron repolarization, altered generation of complex spikes [59,60], and motor defects [61,62]. Restoring Kv3.3 expression in Purkinje neurons of *Kcnc3* knockout mice rescues spiking and motor function [61]. In addition, depending on the mutations of *KCNC3*, Kv3.3 channels can display either loss-of-function or gain-of-function. The p.G592R variant of Kv3.3 shows a gain-of-function phenotype. It results in the degradation of a cell survival protein (Hax-1) that binds to Kv3.3 directly [63]. Other loss-of-function mutations in Kv3.3 that have been identified in human SCA13 patients result in a reduction in Kv3.3 current amplitude and/or Kv3.3 activation voltage [64].

The transcript levels of *Kcnc3* are reduced in mouse models of SCA1, SCA2, and SCA3 (Table 1) [49,65]. In SCA3 mice, the reduction in Kv3.3 transcripts likely contributes to increased excitability of Purkinje neurons and motor impairment [49,66]. Therapeutically, the administration of antisense oligonucleotides targeting the mutated *Atxn3* gene rescues the transcript levels of *Kcnc3*, restores normal Purkinje neuron excitability, and improves the locomotor activity in the early stages of the disease. However, alterations in Purkinje neuron function and motor impairment persist in later disease stages. Meanwhile, a broader reduction in other Kv channels is observed in aged SCA3 mice [49]. Although these reductions do not seem to worsen the Purkinje neuron pathophysiology seen in younger mice, they may contribute to motor dysfunction by altering the function of other brain structures beyond the cerebellum. The detailed mechanism underlying these changes remains to be understood.

The reduction in *Kcnc3* transcript levels has also been reported in mouse models of SCA1 and 2 [65], although little is known regarding the degree of contribution of Kv3.3 to impaired Purkinje neuron firing in these mice. Nevertheless, Kv3.3 dysfunction and dysregulation are associated with multiple cerebellar ataxias [49,62,65,67].

#### 2.2.3. Kv4.3

Kv4.3, encoded by the gene *KCND3*, is another voltage-gated potassium channel highly expressed in cerebellar Purkinje neurons [68]. In humans, mutations in *KCND3* are identified to be the cause of previously designated SCAs: SCA19 and SCA22 (Table 1) [34,35]. The clinical presentation of SCA19/22 includes mild cerebellar ataxia and cognitive impairment. In SCA19/22, the missense mutations in *KCND3* that have been identified include, but are not limited to, p.C317Y, p.P375S, p.V338E, and p.T377M, and are postulated to cause disease due to the loss of function. The mutant Kv4.3 channels show impaired membrane trafficking and are more prone to degradation.

Gain-of-function mutations in Kv4.3 are also implicated in cerebellar ataxia. Recently, a patient presenting with progressive cerebellar ataxia, parkinsonism, cognitive impairment, and iron accumulation in both basal ganglia and cerebellum have been identified to carry a p.R419H mutation in Kv4.3 [69]. Mechanistically, the mutant Kv4.3 channel displays increased outward potassium current and changes in gating properties. In a SCA1 mouse model, increased Kv4.3 current is observed in pre-symptomatic mice, which corresponds to reduced Purkinje neuron firing frequency [5]. Targeting the increased Kv4.3 current with a potassium channel blocker improves firing frequency and motor performance in SCA1 mice [5]. Taken together, both gain- and loss-of-function mutations in Kv4.3 are linked to cerebellar ataxia.

### 2.3. Calcium-Activated Potassium Channels

#### 2.3.1. Large-Conductance Calcium-Activated Potassium (BK) Channels

The large-conductance calcium-activated potassium (BK) channel is encoded by the gene *KCNMA1*. In humans, loss-of-function mutations in *KCNMA1* cause Liang–Wang syndrome (LIWAS), a polymalformation syndrome characterized by global developmental delay and neurological dysfunction (Table 1) [36]. About half of the patients identified with LIWAS show symptoms of ataxia and cerebellar atrophy [36], indicating that loss-of-function BK channel mutations can cause cerebellar ataxia [70].

Downregulation of *Kcnma1* transcripts or BK channel dysfunction has been reported in multiple models of cerebellar ataxia. In a transgenic mouse model of SCA1 (ATXN1[82Q] mice), both the transcripts of *Kcnma1* and the expression of BK channels are decreased [6,9,65]. The decreased level of BK channels is associated with a reduction in the AHP, impaired Purkinje neuron spiking, and motor dysfunction. Significantly, restoring BK channel expression in ATXN1[82Q] mice is able to improve motor performance, suggesting the therapeutic potential of targeting BK channels in SCA1 [6]. Reduced expression of BK channels is also observed in a genetically more precise mouse model of SCA1 (Atxn1^154Q/2Q^) [9,65]. In Atxn1^154Q/2Q^ mice, reduction in BK channel expression is concurrent with irregular Purkinje neuron spiking and impaired motor performance. Applying a BK channel activator (4-chloro-N-(5-chloro-2-cyanophenyl)-3-(trifluoromethyl) benzene-1-sulfonamide, also termed BK-20) is able to rescue Purkinje neuron spiking regularity in Atxn1^154Q/2Q^ mice in vitro [71].

Decreased BK channel expression is also evident in SCA2. In ATXN2[127Q] mice, reduction in the transcripts of *Kcnma1* is accompanied by impaired Purkinje neuron spiking in the early disease stages [67]. Despite the progressive reduction in *Kcnma1* transcripts, regular Purkinje neuron spiking is preserved but at a much lower rate at a later disease stage. The regular but slower Purkinje neuron firing is achieved via the activation of inwardly rectifying potassium (Kir) channels, which may serve as a compensatory mechanism in response to the loss of BK channels [67]. Although restoring BK channel expression or activating remaining BK channels in ATXN2[127Q] mice has not been attempted as a therapeutic strategy, it would be expected that, similar to SCA1 models, this would improve motor function.

Furthermore, a reduction of *Kcnma1* transcript levels is associated with irregular Purkinje neuron spiking in a mouse model of SCA7. Importantly, increasing BK channel expression in the cerebellum of SCA7 mice restores the regularity of Purkinje neuron firing to wild-type levels [7].

It has been shown that in mice, BK channels in Purkinje neurons play a major role in maintaining normal motor coordination. Mice with Purkinje neuron-specific ablation of BK (PN-BK^−/−^) channels display ataxia, the ataxic symptoms of which resemble global *Kcnma1* knockout mice [72]. At the cellular level, Purkinje neurons of PN-BK^−/−^ mice display a depolarized membrane potential, reduced firing frequency, and no change in firing regularity. In addition, the number of complex spikes is greatly reduced in the Purkinje neurons of PN-BK^−/−^ mice, indicating an impairment in both Purkinje neurons and the cerebellar circuit [72,73].

In contrast, gain-of-function mutations of *KCNMA1* are associated with paroxysmal dyskinesia [74], epilepsy, and dystonia. Patients with a p.D434G mutation in BK channels have both paroxysmal dyskinesia [75] and epilepsy [76]. Mechanistically, the p.D434G BK channel variant displays increased calcium sensitivity and enhanced potassium current [74]. In comparison, although the p.N995S variant of BK channels also displays increased potassium current, there is no change in calcium sensitivity. The p.N995S BK channel enhances both channel open probability and duration and hence increases potassium current. In humans, the p.N995S (also annotated as p.N999S and p.N1053S) mutation causes epilepsy but not paroxysmal dyskinesia [76,77]. In mice, knockout of the beta-4 subunit of BK channels results in a gain of function. Beta-4 null mice develop spontaneous seizures, potentially resulting from lowered threshold for the generation of action potentials in hippocampal dentate granule cells [78].

#### 2.3.2. Small-Conductance Calcium-Activated Potassium (SK2) Channels

The brain expresses three types of small-conductance calcium-activated potassium channels. The type 2 small-conductance calcium-activated potassium (SK2) channel, encoded by the gene *KCNN2*, is enriched in cerebellar Purkinje neurons. Recently, exome sequencing has revealed that patients with *KCNN2* mutations exhibit delays in motor, intellectual, and language development (Table 1) [39]. Other symptoms include cerebellar ataxia, tremor, seizures, and extrapyramidal symptoms such as dyskinesia and myoclonus-dystonia [39,79]. Patch-clamp recordings from cells transfected with selected human *KCNN2* mutants reveal a reduction in current density [39], indicating that the loss-of-function of SK2 channels may be responsible for the disease symptoms observed.

Mice harboring a deletion of the *Kcnn2* gene exhibit symptoms of motor dysfunction such as tremors, abnormal gait, and impaired rotarod performance [80]. Genetically targeted silencing of SK channels in the brain of mice also results in profound motor impairment [81]. Modulation of the SK2 channel has shown beneficial effects in a mouse model of SCA2. SCA2-58Q mice show a phenotype of age-dependent onset of motor deficits [82] that is associated with burst firing patterns of Purkinje neurons [11]. Significantly, SK channel activators are able to convert the burst firing pattern back to tonic firing patterns [11]. Oral administration of SK activators also improves motor performance and delays Purkinje neuron degeneration in SCA2-58Q mice. Furthermore, a loss of function of the SK2 channel has been described as the underlying abnormality in mouse models of tremor [80,83]. No gain-of-function mutations in *KCNN2* have been reported.

### 2.4. Voltage-Gated Calcium Channels

#### 2.4.1. Cav2.1

*CACNA1A* encodes the P/Q-type voltage-gated calcium channel Cav2.1, which is highly enriched at synaptic terminals and in Purkinje neurons [84]. Mutations in *CACNA1A* are associated with SCA6 and episodic ataxia type 2 (EA2) (Table 1) [85,86].

In SCA6, a CAG repeat expansion in *CACNA1A* results in a poly-glutamine tract at the C-terminus of Cav2.1 [85]. Patients affected with SCA6 have 20-33 CAG repeats in the *CACNA1A* gene, with slowly progressive and late-onset ataxia symptoms [87,88]. A SCA6 mouse model captures the late-onset disease phenotype as seen in human patients. Heterozygous SCA6^84Q/+^ mice develop motor impairment at 19 months of age, whereas homozygous SCA6^84Q/84Q^ mice have an earlier disease onset at 7 months of age [10,89]. Surprisingly, although the CAG repeat expansion is in the gene encoding Cav2.1, the function of the calcium channel itself in Purkinje neurons does not appear to be impaired [89,90]. The exact disease mechanism accounting for the late onset of symptoms in SCA6 remains unknown.

Mutations in the *CACNA1A* gene are also associated with EA2, characterized by episodes of recurrent ataxic symptoms in humans [91,92]. One widely used mouse model of EA2 is the tottering mouse. Tottering mice, which carry a p.P601L missense mutation in the *Cacna1a* gene [93], display ataxic symptoms such as paroxysmal attacks of motor dysfunction and abnormal eye movements [94,95]. Purkinje neurons of tottering mice exhibit firing abnormalities [94,96]. The irregular Purkinje neuron spiking is associated with a reduction in calcium current density [97].

Gain-of-function mutations in Cav2.1 are also associated with abnormal Purkinje neuron firing. The p.S218L missense mutation in *Cacna1a* results in an increase in calcium influx into Purkinje neurons, which lowers the threshold for action potential generation, causing a disrupted firing pattern [98]. Mice with the p.S218L mutation display symptoms of ataxia [99], indicating that both increased and decreased calcium influx can be associated with ataxia.

#### 2.4.2. Cav3.1

Cav3.1, encoded by the gene *CACNA1G*, is a T-type calcium channel highly expressed in Purkinje neurons. In humans, the p.R1715H missense mutation in the *CACNA1G* gene causes SCA42, symptoms of which include predominantly gait instability (Table 1) [100]. The p.R1715H mutation results in a positive shift in the voltage dependence of Cav3.1 activation [101]. Zonisamide, a T-type calcium channel blocker, reverses the abnormal voltage dependence of current activation of the mutant channel close to wild-type levels in HEK293T cells [102]. In addition, reduced transcript levels of *Cacna1g* have been reported in other SCA mouse models, including SCA1, 2, and 7 [7,9,65,103]. Mice lacking Cav3.1 display impaired motor coordination, Purkinje neuron loss, and cerebellar atrophy [104].

Gain-of-function mutations in Cav3.1 are associated with childhood-onset cerebellar atrophy with additional clinical features, including cognitive impairment and variable facial dysmorphism, microcephaly, and epilepsy [105].

### 2.5. Other Calcium Channels and Calcium Pumps

#### 2.5.1. TRPC3

TRPC3 is one of the family members of the transient receptor potential (TRP) superfamily of ion channels that are highly enriched in cerebellar Purkinje neurons [106]. Mutations in the *TRPC3* gene cause SCA41 in humans (Table 1). Recently, a p.R762H missense mutation in *TRPC3* has been identified in a patient with a progressive imbalance and ataxic gait [43]. The p.R762H variant induces significant neuronal death, indicating a gain-of-function mechanism. Moonwalker (*Mwk*) mice, harboring a gain-of-function mutation in *Trpc3*, display coordination defects and Purkinje neuron degeneration [107]. Mechanistically, in *Mwk* mice, the TRPC3 channel displays prolonged channel opening upon activation [107]. Patch clamp recordings from *Mwk* mice show that a greater number of Purkinje neurons are inactive compared to wild-type neurons. Additionally, the Purkinje neurons of *Mwk* mice that are active fire at a greater frequency than wild-type neurons, indicating the inactive ones are likely in depolarization block [108].

#### 2.5.2. IP_3_R1

IP_3_R1, or inositol 1,3,5-trisphophate (IP_3_) receptor type 1, is encoded by the gene *ITPR1*. IP_3_R1 functions as a ligand-gated ion channel and mediates calcium release from the endoplasmic reticulum. Deletions or mutations in *ITPR1* lead to SCA15 in humans, the clinical presentation of which is characterized by adult-onset and slow progression of cerebellar gait ataxia (Table 1) [97]. Missense mutations in *ITPR1*, including p.V494I and p.P1059L, have also been identified in families with SCA15 [44,109]. Missense mutations in *ITPR1* are also associated with SCA29 in humans (Table 1) [45]. Patients with SCA29 present with infant-onset slowly progressive ataxia, delayed motor development, and mild cognitive impairment [110]. Furthermore, patients with autoantibodies against IP_3_R1 also develop cerebellar ataxia [111]. The underlying mechanism of SCA15 and SCA29 pathology is likely due to suppressed cytosolic calcium signaling, which results from the loss-of-function of IP_3_R1.

Studies in mice show that increased IP_3_R1 activity may also contribute to cerebellar ataxia. It has been shown that in SCA2-58Q mice, mutant ATXN2 interacts with IP_3_R1, leading to increased activation sensitivity of IP_3_R1 and enhanced calcium release from the endoplasmic reticulum [82]. Aberrant calcium signaling also contributes to the pathogenesis of SCA3. Similar to the mutant ATXN2 protein, mutant ATXN3 (Q77 and Q127) can also bind to IP_3_R1 and increase the sensitivity of IP_3_R1 to IP_3_ [112].

#### 2.5.3. PMCAs

The calmodulin-activated plasma membrane calcium-ATPases (PMCAs), especially the brain-enriched isoforms, type 2 (PMCA2) and type 3 (PMCA3), function to maintain cellular calcium homeostasis by pumping calcium out of the cell [113]. In humans, mutations in the gene encoding PMCA2 are linked to hearing loss. Recently, a missense mutation in PCMA2, V1143F, has been identified in a patient with congenital cerebellar ataxia but no sign of deafness (Table 1) [46]. The mutation causes a loss-of-function mutation of PMCA2, leading to impaired calcium ejection from the cytoplasm. In mice, PMCA2 is highly expressed in various tissue types, including cerebellar Purkinje neurons. *Atp2b2* (encodes PMCA2) knockout mice exhibit overt cerebellar ataxia [114]. Recordings from Purkinje neurons of *Atp2b2* knockout mice reveal slower and irregular firing and a more hyperpolarized membrane potential [115]. Missense mutations in PMCA3 are associated with ataxia. The missense mutation G1107D in *ATP2B3* has been identified in patients with X-linked congenital cerebellar ataxia [47,116] (Table 1). Functional analysis of the mutant PMCA3 demonstrates impaired calcium pumping ability, indicating a loss-of-function mutation. Taken together, both PMCA2 and PMCA3 play a role in ataxia pathogenesis.

## 3. Emerging Therapies for Cerebellar Ataxia Impinge on Ion Channels

### 3.1. Non-Pharmacological Approaches in Cerebellar Ataxia

#### 3.1.1. Rehabilitation

Currently, there is no definitive treatment for cerebellar ataxia. Most of the current clinically used treatments rely on strategies for rehabilitation that include physical therapy, occupational therapy, and speech therapy. Rehabilitation in cerebellar ataxia aims to improve balance and coordination in patients through intensive exercise [117]. It has been shown that rehabilitation training can improve the impairment measured on an ataxia rating scale [118,119,120], with evidence that the improvement can persist for up to 1 year. The exact mechanism of how exercise improves motor function in cerebellar ataxia patients with progressive cerebellar atrophy is still unknown. In SCA6^84Q/84Q^ mice, exercise has been shown to improve Purkinje neuron firing defects and thereby improve ataxia, potentially through increasing cerebellar expression of brain-derived neurotrophic factor [12]. So far, this is the only evidence showing that exercise can modify Purkinje neuron firing in cerebellar ataxia. It is likely that exercise modifies potassium channel expression and function to mediate this improvement in spiking.

#### 3.1.2. Gene Suppression Strategies

Oligonucleotide-based therapy is an emerging field aimed at treating genetic disorders. Oligonucleotide-based therapy that has been implicated in treating cerebellar ataxias includes antisense oligonucleotide and RNA-based therapy [121]. Antisense oligonucleotides (ASOs) are small, single-stranded DNA molecules that have the capability to reduce the expression of specific proteins by binding to complementary mRNA transcripts [122]. The therapeutic effects of ASOs have been shown in several mouse models of cerebellar ataxia, including SCA1, SCA2, SCA3, and SCA13 [63,123,124,125]. In the Knockin mouse model of SCA1 (Atxn1^154Q/2Q^) that displays ataxia and premature lethality, ASO delivery to the right lateral ventricle improves motor performance and prolongs survival [123]. Injection of ASOs into the cerebellum of SCA2 mice can successfully reduce Atxn2mRNA and protein levels in Purkinje neurons, resulting in delayed motor symptom onset [124]. In addition, ASO treatment improves Purkinje cell firing in parallel with behavioral improvement. Longitudinal ASO delivery targeting mutant *Atxn3* in SCA3 mice restores transcript levels of Kv3.3 and Kv1.6 associated with improved Purkinje neuron firing and motor performance [49]. In the Kv3.3-G592R mouse model of SCA13, intracerebroventricular injection of ASOs targeting mutant *Kcnc3* mRNA restores motor performance to a level comparable with wild-type controls [63].

Gene silencing through viral-mediated RNAi is an alternative method to ASOs to reduce the level of targeted proteins. Intracerebellar injection of an adeno-associated virus that expresses short hairpin RNAs against ATXN1 has been shown to significantly improve motor coordination in SCA1 mice [126,127].

### 3.2. Pharmacological Approaches in Cerebellar Ataxia

Treatment for cerebellar ataxia is needed. Few medications have proven effective in managing symptoms of cerebellar ataxia. The majority of agents that have been proposed for treatment have been unsuccessful in clinical trials. Here, we discuss the clinical data and the mechanism of action of the agents that are currently prescribed for treating cerebellar ataxia. These include omaveloxolone, 4-aminopyridine, and chlorzoxazone-baclofen. Importantly, many of these agents target ion channels, further illustrating the therapeutic potential of modulating ion channels for cerebellar ataxia.

#### 3.2.1. Omaveloxolone

Recently, the US FDA approved omaveloxolone as the first and only therapy for Friedreich ataxia, an autosomal recessive cerebellar ataxia. Clinical symptoms in patients with Friedreich ataxia include gait and limb ataxia, discoordination, loss of lower limb reflexes, dysarthria, diabetes, cardiomyopathy, scoliosis, and vision loss [128]. Friedreich ataxia results from a GAA repeat expansion in the *FXN* gene, which encodes the protein frataxin that is localized to mitochondria [129]. The function of frataxin is not entirely understood, but it has been shown to be involved in iron–sulfur cluster assembly [130]. A GAA repeat expansion in *FXN* results in a reduction of functional frataxin protein. Consequences of frataxin deficiency include mitochondrial dysfunction, increased sensitivity to oxidative stress, and dysregulation of iron-sulfur cluster assembly [131,132,133]. Friedreich ataxia is, therefore, considered to be a mitochondrial disease.

The proposed mechanism of action of omaveloxolone is the activation of the nuclear factor erythroid 2-related factor 2 (NRF2) [134,135]. Under normal conditions, oxidative stress induces NRF2 translocation to the nucleus and increases the expression of antioxidant genes. In Friedreich ataxia, however, NRF2 fails to respond to oxidative stress [134]. The impaired antioxidant defense system may contribute to neurodegeneration in Friedreich ataxia since neurons are sensitive to cellular redox status [136]. Omaveloxolone, as a potent activator of NRF2, has been shown to restore mitochondrial function in multiple mouse models of Friedreich ataxia. In clinical trials, patients receiving omaveloxolone show significant improvement in motor performance than patients receiving a placebo [137]. Given omaveloxolone’s ability to rescue antioxidant defense systems, it may also be therapeutically beneficial in other cerebellar ataxias that are associated with mitochondrial dysfunction.

SCA28 is the only known dominantly inherited cerebellar ataxia caused by defects in a mitochondrial protein [138]. The mutated gene responsible for SCA28, *AFG3L2*, encodes a subunit of a human mitochondrial ATPase associated with various cellular protease activities (m-AAA) [139]. Members of the m-AAA protease family participate in protein quality control within mitochondria [140,141]. Clinically, patients affected by SCA28 demonstrate slowly progressive gait and limb ataxia. In a SCA28 mouse model that is haploinsufficient for *Afg3l2*, progressive loss of cerebellar Purkinje neurons, thinning of the cerebellar molecular layer, and impaired motor performance are noted [138]. Purkinje neuron neurodegeneration is likely to be a result of enhanced cytoplasmic calcium concentrations due to defects in mitochondrial calcium buffering capacity [140]. Reducing cellular calcium influx is able to improve ataxia in the *Afg3l2* haploinsufficient mice [142]. Additionally, Purkinje neurons in mice that are heterozygous for the p.M665R-*Afg3l2* allele appear to be more excitable than wild-type mice [143].

Mitochondrial defects have also been shown to contribute to disease in autosomal recessive spastic ataxia of Charlevoix–Saguenay (ARSACS), a childhood-onset disorder with pyramidal spasticity and cerebellar ataxia. ARSACS results from mutations in the *SACS* gene encoding the sacsin protein, which has been shown to regulate the connectivity of the mitochondrial network [144]. Purkinje neurons of *Sacs* knockout mice demonstrate disordered dendritic morphology and slow but regular firing [145] before degeneration.

Taken together, it is likely that impairments in the mitochondrial network can lead to alterations in Purkinje neuron intrinsic membrane excitability and promote Purkinje neuron degeneration. Omaveloxolone, therefore, may have a broader therapeutic potential beyond Friedreich ataxia.

#### 3.2.2. 4-Aminopyridine

4-aminopyridine (4-AP) functions as a non-selective voltage-gated potassium (Kv) channel blocker. Currently, it is prescribed for patients with EA2, multiple sclerosis, and for the treatment of symptomatic downbeat nystagmus. Several studies have shown that 4-AP reduces ataxia attack frequency and decreases attack duration in EA2 patients [146,147]. In a mouse model of EA2, abnormal Purkinje neuron spiking is associated with reduced calcium current through the mutant Cav2.1 channel. 4-AP has been shown to restore the precision of Purkinje neuron pacemaking via the prolongation of action potentials and increasing the amplitude of the AHP [148]. 4-AP appears to restore Purkinje neuron pacemaking with a mechanism similar to Chlorzoxazone, a calcium-activated potassium channel (Kca) activator. By inhibiting Kv channels, 4-AP appears to activate Kca channels. Besides EA2, 4-AP has been shown to reduce the frequency and/or severity of ataxia symptoms in patients with SCA27B [29]. Chronic administration of 4-AP to SCA6^84Q/84Q^ mice restores the precision of Purkinje neuron spiking and improves motor performance [10]. Acute treatment of early symptomatic SCA1 mice with 3,4-diaminopyridine, an analog of 4-AP, restores normal Purkinje neuron firing and improves motor performance. Chronic treatment of SCA1 mice with 3,4-diaminopyridine not only normalizes firing frequency and improves motor function but also partially protects against neuronal degeneration [5].

Blockade of Kv channels by 4-AP as a means to increase neuronal conduction has been used as a therapeutic approach in multiple sclerosis (MS). Clinically, MS patients benefit from 4-AP with improved motor function and vision [149,150]. Studies from experimental mouse models of MS show that demyelination causes an axonal redistribution of potassium channels (especially Kv1.1 and Kv1.2) [151] that are normally clustered near the nodes of Ranvier [152]. Demyelination also leads to enhanced exposure of potassium channels. The misdistribution and increased exposure of potassium channels cause an increase in the threshold for successful action potential generation, which impairs action potential progression and results in motor dysfunction in MS [153]. It has been shown that by blocking potassium channels with 4-AP, action potential conduction is restored.

#### 3.2.3. Chlorzoxazone and Baclofen

Chlorzoxazone (CHZ) is an FDA-approved skeletal muscle relaxant used to treat muscle spasms and pain. Although the exact mechanism of action is unknown, it activates SK and BK channels [154,155]. Clinically, CHZ has been suggested to improve eye movements and visual acuity in patients with cerebellar downbeat nystagmus [156]. In mouse studies, CHZ improves Purkinje neuron firing and motor performance in multiple mouse models of cerebellar ataxia. Intraperitoneal injection of CHZ in SCA2-58Q mice converts irregular bursting Purkinje neuron firing patterns into regular tonic firing in vivo [157]. In the same study, SCA2-58Q mice CHZ also improved performance on a beam-walk test. In EA2 mice, CHZ restores irregular Purkinje neuron firing in vitro and significantly reduces the frequency and duration of ataxia attacks when given orally [148].

Baclofen is another FDA-approved skeletal muscle relaxant that has been used to treat muscle spasms, stiffness, and pain in patients with multiple sclerosis and/or spinal cord injury/disease. Intrathecal baclofen (ITB) infusion is accepted as a treatment for spasticity (increased rigidity of muscle secondary to injury), where it has been shown to improve positioning and decrease muscle tone in targeted patients [158]. One study has shown ITB treatment has beneficial effects in patients with SCA3, SCA7, and Friedreich ataxia who have spasticity and muscle spasms [159]. From a molecular standpoint, baclofen functions as a GABA_B_ receptor agonist in the central nervous system [160]. In the cerebellum, GABA_B_ receptors are highly enriched at pre- and post-synaptic terminals [161,162]. It has been shown that activation of GABA_B_ receptors modulates neuronal excitability via activation of G protein-gated inwardly rectifying K^+^ (GIRK or Kir3) channels in cerebellar Purkinje neurons [163]. Under normal conditions, inwardly rectifying K^+^ (K_ir_) channels function in maintaining resting membrane potential in neurons [164]. However, their function may be altered in the disease. In a transgenic mouse model of SCA2, a significant number of non-firing Purkinje neurons is associated with loss of BK and Kv3.3 channels expression at an early disease stage. However, Purkinje neuron firing in the mutant mice is restored at a later disease stage, although at a significantly slower frequency. It has been found that rescued slow Purkinje neuron firing frequency is a result of a novel AHP produced by K_ir_ channels [67], indicating that K_ir_ channels may have a compensatory role in restoring Purkinje neuron physiology when the AHP mediated by BK channels is impaired. A similar loss of the AHP is observed in SCA1 mouse models [6,9]. Given Baclofen’s ability to create a novel AHP, a combination of CHZ and baclofen was identified to restore the Purkinje neuron AHP in these models of SCA1 [8,9]. Administration of a combination of baclofen and CHZ in vivo improves motor impairment in several models of SCA1 [8,9].

Clinically, CHZ and baclofen are often prescribed together to patients with cerebellar ataxias. In patients with SCA1, SCA2, SCA6, SCA8, and SCA13, co-administered CHZ and baclofen are suggested to improve motor impairment [8].

### 3.3. Other Approaches

#### Cerebellar Stimulation: Deep Brain Stimulation and Non-Invasive Cerebellar Stimulation

Deep brain stimulation (DBS) is currently used to treat patients with Parkinson’s disease, dystonia, and tremors. Although the use of DBS in patients with ataxia is limited, ataxia patients may potentially benefit from this therapy. Several studies have shown that DBS can relieve tremors and/or dystonia associated with ataxia. One study showed that high-frequency thalamic ventralis intermedius nucleus (VIM) stimulation and low-frequency stimulation of subthalamic projections attenuated tremors in two patients with SCA27 with no effect on ataxia [165]. In another study, VIM stimulation improved tremor in patients with SCA2 and idiopathic tremor-ataxia syndrome, whereas Globus pallidus interna (Gpi) DBS improved dystonia in one SCA17 patient and one patient with a senataxin mutation, with no evidence of improvement in ataxia-related symptoms [166]. Long-term VIM stimulation regularized the gait cycle and improved tremor in three patients with Fragile X-associated tremor/ataxia syndrome (FXTAS) [167]. However, the outcome of VIM stimulation was reported as poor in another FXTAS patient [166]. Dentate nucleus DBS (DN DBS) was also shown to effectively alleviate cerebellar tremors in patients with SCA3 or post-lesion ataxia [168]. In the same patients, DN DBS moderately improved ataxia symptoms, although the effect was not significant. Despite the lack of evidence for the improvement of ataxia via DBS in human studies, DBS targeting the cerebellum has shown promising therapeutic effects in mouse models of ataxia. DBS stimulation of the interposed cerebellar nucleus induces long-lasting motor benefits and significantly improves motor performance in mouse models of ataxia [169,170].

Another promising therapeutic modality for cerebellar disorders is non-invasive cerebellar stimulation, which includes transcranial magnetic stimulation (TMS) and transcranial direct current stimulation (tDCS). The therapeutic effects of non-invasive cerebellar stimulation on 151 cerebellar ataxia patients from eight studies were summarized in the systemic review by Di Nuzzo et al. [171].

A detailed mechanism of how cerebellar stimulation improves motor dysfunction remains to be understood. A recent modeling study suggests that cerebellar DBS can restore the reduced inhibitory input resulting from Purkinje neuron degeneration in ataxic mice [172], indicating cerebellar stimulation has the potential to modulate synaptic activity and downstream neuronal pathways for motor control. Proper synaptic activity at both pre- and postsynaptic levels relies heavily on ion channels [173]. Additionally, it has been proposed that low-frequency stimulation likely excites neurons at both soma and axon levels, whereas high-frequency stimulation exhibits inhibitory effects [174], potentially via the induction of depolarization block via inactivation of voltage-gated ion channels. Therefore, cerebellar stimulation may elicit its therapeutic effects through the modulation of synaptic ion homeostasis.

## 4. Discussion: Is There a “Levodopa” for Cerebellar Ataxia?

Cerebellar ataxias are a group of devastating diseases that severely affect patients’ quality of life. Unfortunately, effective treatments for cerebellar ataxia are limited. The recently FDA-approved medication for Friedreich ataxia, omaveloxolone, is an exciting development and holds promise for the development of other therapeutic modalities in the future. A therapy that could benefit a broader range of cerebellar ataxia patients and with a larger effect size is still needed.

Based on current studies in ataxia mouse models, it is clear that abnormal Purkinje neuron spiking is associated with impaired motor function. Treatments aimed at restoring normal Purkinje neuron spiking have been shown to improve motor performance. Normal Purkinje neuron spiking is tightly dependent on membrane excitability regulated by ion channels. Changes in ion channel expression and/or function in disease conditions are likely to impair Purkinje neuron intrinsic membrane excitability, leading to irregular and/or slow Purkinje neuron firing patterns. The proper ion channel function is, therefore, vital for regulating Purkinje neuron spiking and, hence, precise motor coordination.

**Figure 1:**
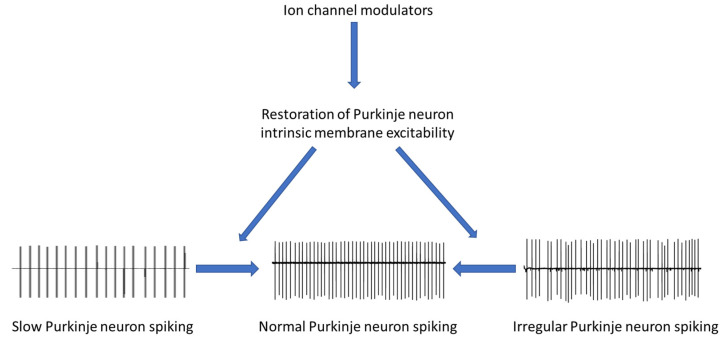
Scheme showing Purkinje neuron pathophysiology in cerebellar ataxia and how modulation of Purkinje neuron intrinsic membrane excitability is a potential therapy for cerebellar ataxia.

Mutations in a variety of genes have been linked to cerebellar ataxias. Recent studies in mouse models of ataxia of differing etiology suggest that a shared feature among cerebellar ataxias is defects in ion channels (altered expression and/or function). Clinically, agents that modulate ion channels may improve symptoms in ataxia patients. For example, the non-selective potassium channel blocker 4-AP appears to improve motor performance in patients with EA2 and SCA27B. Co-administration of chlorzoxazone and baclofen may improve symptoms in patients with SCA1, SCA2, SCA6, SCA8, and SCA13. In ataxia mouse models, these ion channel modulators have also been shown to improve motor function. Mechanistically, improvements in motor behavior in mice by ion channel modulators are associated with improvements in abnormal Purkinje neuron spiking. From a therapeutic perspective, ion channel modulators that can rescue irregular Purkinje neuron spiking or increase Purkinje neuron firing frequency when it is reduced would theoretically have the ability to restore normal Purkinje neuron intrinsic membrane excitability and normalize spiking in disease states and thus improve motor function. Therapies for cerebellar ataxia should, therefore, aim to restore Purkinje neuron intrinsic membrane excitability by normalizing Purkinje neuron spiking regularity when it is irregular and improving Purkinje neuron firing rate when the firing frequency is reduced. Since ion channels are crucial regulators of Purkinje neuron intrinsic membrane excitability, we propose that agents targeting ion channels have the potential to be a shared therapy for cerebellar ataxia independent of the disease-causing gene (Figure 1).

### 4.1. Voltage-Gated Sodium Channels

Both Nav1.6 and Nav1.1 play important roles in the generation of the Purkinje neuron action potential upstroke. Mutations in the genes encoding Nav1.6 and Nav1.1 produce ataxia symptoms in both humans and mice. Specifically, both gain- and loss-of-function mutations in the gene encoding Nav1.6 and loss-of-function mutations of the gene encoding Nav1.1 can result in ataxia. Therefore, activating or inhibiting these sodium channels and tailored to the underlying mechanism may have therapeutic potential in ataxia. It is important to note that although the reduction in sodium current is associated with ataxia, the degree of activation of sodium channels should be tightly controlled. Increased sodium channel activity has been shown to play a role in contributing to neurodegeneration in multiple sclerosis [175]. It has been shown that sustained sodium influx can overwhelm the function of the Na/K ATPase, leading to the accumulation of sodium in axons and thereby activating the Na/Ca exchanger [176]. Activation of the Na/Ca exchanger moves sodium outside of axons at the expense of elevating intra-axonal calcium levels, which contributes to axonal degeneration [177]. Increased calcium concentrations can have detrimental effects on neurons. Separately, an increased inward calcium current can also cause ataxia [98]. Similarly, the dose of sodium channel inhibitors in treating gain-of-function-mutation-associated ataxia should also be tightly controlled as the sodium current is crucial for the generation of action potentials.

### 4.2. Voltage-Gated Potassium Channels

Defects in voltage-gated potassium channels, including Kv1 family members, Kv3.3, and Kv4.3, are associated with cerebellar ataxia. 4-AP, a non-selective voltage-gated potassium channel inhibitor, has shown promising therapeutic effects in certain ataxia patient populations, including patients with EA2 and SCA27B. 4-AP can restore Purkinje neuron spiking regularity without affecting firing frequency in mouse models of SCA6 and EA2. 3,4-diaminopyridine, an analog of 4-AP, has been shown to improve the reduced Purkinje neuron firing frequency in a mouse model of SCA1 [5]. However, one potential downside of 4-AP being non-selective is that it blocks different types of Kv channels at similar concentrations [178]. Importantly, loss-of-function mutations in certain Kv channels cause ataxia. Loss-of-function mutations of Kv3.3 and Kv4.3 result in SCA13 and SCA19/22, respectively. Reduced transcript levels of the gene encoding Kv1.6 is evident in a mouse model of SCA3. Loss-of-function mutations in Kv1.1 and Kv1.2 are also associated with ataxia phenotypes. Therefore, when considering 4-AP as a shared therapy for cerebellar ataxias, one must take into account that 4-AP has the potential to cause ataxia. Furthermore, a clinical trial examining the short-term effect of 4-AP on patients with SCA1, SCA3, and SCA6 showed no change in the score of an ataxia rating scale compared to a placebo [179].

### 4.3. Calcium-Activated Potassium Channels

Activation of BK channels can improve Purkinje neuron spiking irregularity and increase Purkinje neuron firing frequency. In Atxn1^154Q/2Q^ mice, irregular Purkinje neuron spiking is associated with motor impairment. BK-20, a BK channel activator, significantly improves the regularity of Purkinje neuron spiking in vitro. Additionally, BK-20 increases firing frequency in Atxn1^154Q/2Q^ Purkinje neurons [71]. We, therefore, speculate that in ataxia, where phenotype results from either irregular or slow Purkinje neuron firing, activating BK channels would help normalize firing. However, the dose of BK channel activators should be tightly controlled as BK channel overactivation is associated with paroxysmal dyskinesia, epilepsy, and dystonia [74].

SK2 channels may also be targeted for treating cerebellar ataxia. Although mutations in SK2 channels have only been identified in a few patients with ataxia, agents activating SK2 channels have been shown to improve Purkinje neuron firing regularity and motor performance in mouse models of cerebellar ataxia. The effect of SK2 activation on Purkinje neuron firing frequency varies. For example, SK2 activators increase Purkinje neuron firing frequency in vivo but decrease firing frequency in vitro in Cav2.1-S218L mice [98]. Additionally, higher concentrations of chlorzoxazone, an SK2 channel activator, significantly decrease Purkinje neuron firing frequency in Atxn1^154Q/2Q^ mice [9].

### 4.4. Calcium Channels and Calcium Pumps

Calcium channels, including Cav2.1, Cav3.1, TRPC3, and IP_3_R1, and calcium pumps, including PMCA2 and PMCA3, have been implicated in cerebellar ataxia in both humans and mice. Both gain- and loss-of-function mutations in these calcium channels can cause abnormal Purkinje neuron spiking and motor dysfunction in mice, indicating a vital role of calcium currents and intracellular calcium concentrations in maintaining Purkinje neuron intrinsic membrane excitability. Similarly, mutations in other calcium channels (for example, calcium-release activated calcium channels) that lead to increased or decreased calcium influx may, therefore, also be associated with Purkinje neuron pathophysiology and ataxia. Furthermore, the proper function of calcium-activated potassium channels depends on appropriate calcium flux and intracellular calcium concentrations. Taken together, targeting calcium channels may represent another therapeutic approach to treating cerebellar ataxia.

## 5. Conclusions

Ion channel dysfunction is a shared disease mechanism in cerebellar ataxias despite the various disease-causing genes. Dysfunction in ion channels disrupts Purkinje neuron intrinsic membrane excitability, which impairs Purkinje neuron spiking and motor function. Therefore, we propose that targeting Purkinje neuron intrinsic membrane excitability holds promise to restore spiking irregularity and increase slowed Purkinje neuron firing. Therapies aiming at restoring normal Purkinje neuron intrinsic membrane excitability, therefore, have the greatest potential to be the “levodopa” for cerebellar ataxia.

The long-term benefits and potential complications of the use of ion channel modulators in cerebellar ataxia are currently unknown. In addition, in Parkinson’s disease, levodopa becomes less effective as new symptoms develop and the disease progresses [180]. Similarly, in cerebellar ataxia, it is likely that combining ion channel modulator therapy with other treatments may be needed for the long-term management of progressive symptoms.

## Data Availability

Data sharing not applicable.
